# Basolateral Mg^2+^ Extrusion via CNNM4 Mediates Transcellular Mg^2+^ Transport across Epithelia: A Mouse Model

**DOI:** 10.1371/journal.pgen.1003983

**Published:** 2013-12-05

**Authors:** Daisuke Yamazaki, Yosuke Funato, Jiro Miura, Sunao Sato, Satoru Toyosawa, Kazuharu Furutani, Yoshihisa Kurachi, Yoshihiro Omori, Takahisa Furukawa, Tetsuya Tsuda, Susumu Kuwabata, Shin Mizukami, Kazuya Kikuchi, Hiroaki Miki

**Affiliations:** 1 Department of Cellular Regulation, Research Institute for Microbial Diseases, Osaka University, Suita, Japan; 2 Division for Interdisciplinary Dentistry, Dental Hospital, Osaka University, Suita, Japan; 3 Department of Oral Pathology, Graduate School of Dentistry, Osaka University, Suita, Japan; 4 Department of Pharmacology, Graduate School of Medicine, Osaka University, Suita, Japan; 5 Laboratory for Molecular and Developmental Biology, Institute for Protein Research, Osaka University, Suita, Japan; 6 PRESTO, Japan Science and Technology Corporation, Kawaguchi, Japan; 7 CREST, Japan Science and Technology Corporation, Kawaguchi, Japan; 8 Department of Applied Chemistry, Graduate School of Engineering, Osaka University, Suita, Japan; 9 Division of Advanced Science and Biotechnology, Graduate School of Engineering, Osaka University, Suita, Japan; Radboud University Medical Center, Netherlands

## Abstract

Transcellular Mg^2+^ transport across epithelia, involving both apical entry and basolateral extrusion, is essential for magnesium homeostasis, but molecules involved in basolateral extrusion have not yet been identified. Here, we show that CNNM4 is the basolaterally located Mg^2+^ extrusion molecule. CNNM4 is strongly expressed in intestinal epithelia and localizes to their basolateral membrane. CNNM4-knockout mice showed hypomagnesemia due to the intestinal malabsorption of magnesium, suggesting its role in Mg^2+^ extrusion to the inner parts of body. Imaging analyses revealed that CNNM4 can extrude Mg^2+^ by exchanging intracellular Mg^2+^ with extracellular Na^+^. Furthermore, *CNNM4* mutations cause Jalili syndrome, characterized by recessive amelogenesis imperfecta with cone-rod dystrophy. CNNM4-knockout mice showed defective amelogenesis, and CNNM4 again localizes to the basolateral membrane of ameloblasts, the enamel-forming epithelial cells. Missense point mutations associated with the disease abolish the Mg^2+^ extrusion activity. These results demonstrate the crucial importance of Mg^2+^ extrusion by CNNM4 in organismal and topical regulation of magnesium.

## Author Summary

Magnesium is an essential element for living organisms. Its absorption occurs at the intestine through the barrier comprised of epithelial cells. In this process, transcellular Mg^2+^ transport across epithelia, involving both entry from one side and extrusion from the other side, is important. Previous studies have revealed the role of Mg^2+^-permeable channel protein in Mg^2+^ entry into the epithelial cells. However, the identity of proteins involved in Mg^2+^ extrusion to the inner parts of body has remained unknown. Mice genetically engineered not to express CNNM4, which localizes to the epithelial membrane facing to the inner parts of body, show hypomagnesemia due to the defect in magnesium absorption. Functional analyses using culture cells directly reveal that CNNM4 can extrude intracellular Mg^2+^ to the outside of cells. These results indicate that CNNM4 mediates transcellular Mg^2+^ transport across the intestinal epithelia. Furthermore, we also show that these CNNM4-lacking mice also have a defect in amelogenesis, which is consistent with the disease symptoms of Jalili syndrome that is known to be caused by mutations in the *CNNM4* gene.

## Introduction

Magnesium is an essential element involved in a wide variety of biological activities. Homeostasis of the magnesium level is strictly regulated by intestinal absorption and renal reabsorption, in which epithelia function as a barrier that permits selective and regulated transport of Mg^2+^ from apical to basolateral surfaces. Genomic analyses of familial cases of hypomagnesemia have identified key molecules directly involved in these processes. *CLDN16*, encoding claudin-16/paracellin-1, and *CLDN19*, encoding claudin-19, are mutated in recessive familial hypomagnesemia with hypercalciuria and nephrocalcinosis [1], [2]. These genes are highly expressed in the thick ascending limb of Henle's loop in the kidney and encode tight junction proteins, which form a cation-selective paracellular channel and drive the flux of Mg^2+^ between adjacent epithelial cells [3]. Another key molecule is TRPM6; mutations of *TRPM6* cause recessive hypomagnesemia with secondary hypocalcemia [4], [5].

TRPM6 is a member of the transient receptor potential melastatin-related (TRPM) protein family and constitutes a Mg^2+^-permeable ion channel that localizes to the apical membrane of epithelial cells in the intestine and kidney [6]. In addition, it has also been shown that TRPM7, a close relative of TRPM6, plays an essential role in magnesium homeostasis in mice [7]. Therefore, TRPM6/TRPM7 plays a primary role in the apical entry of Mg^2+^ into cells, which is the first step in transcellular Mg^2+^ absorption across the epithelial barrier, another major Mg^2+^ transport pathway. To accomplish Mg^2+^ absorption, epithelial cells need to extrude Mg^2+^ via their basolateral membrane by opposing the inward-oriented driving force on Mg^2+^ imposed by the electrical membrane potential. Such a transcellular Mg^2+^ transport mechanism, involving both apical entry and basolateral extrusion, is evolutionarily conserved from *Caenorhabditis elegans* [8], [9], but molecules involved in basolateral Mg^2+^ extrusion have not been identified.

Ancient conserved domain protein/cyclin M (CNNM) constitutes a family of 4 integral membrane proteins that possess an evolutionarily conserved but uncharacterized domain from bacteria [10]. Recent genomic analyses have revealed a link between *CNNM* genes and magnesium homeostasis. Several single nucleotide polymorphisms in *CNNM* genes are associated with the serum magnesium level [11] and mutations in *CNNM2* cause familial dominant hypomagnesemia [12]. The bacterial ortholog of these proteins in *Salmonella*, CorC, has been suggested to participate in Mg^2+^ efflux [13], while ectopically expressed CNNM2 in *Xenopus* oocytes showed voltage-dependent transport of several divalent cations, including Mg^2+^ [14]. Moreover, expression of a splice-variant of *CNNM2* could restore the growth of a Mg^2+^-deficient *Salmonella* strain [15]. However, a study on CNNM2 expressed in HEK293 cells showed that it mediates a Na^+^ current [12]. Therefore, the importance of CNNMs in Mg^2+^ transport still remains unknown. Moreover, it has been reported that mutations in *CNNM4* cause Jalili syndrome, which is characterized by recessive amelogenesis imperfecta (AI) and cone-rod dystrophy (CRD) [16], [17]. However, the molecular mechanism that links CNNM4 dysfunction to these pathological conditions and its relationship with magnesium homeostasis remain to be determined.

In this study, we generated CNNM4-knockout mice; these mice showed defects in amelogenesis and intestinal Mg^2+^ absorption. Endogenous CNNM4 is highly expressed in the mature ameloblasts and intestinal epithelia, and localizes at their basolateral membrane. Functional analyses at the molecular and organismal levels revealed a common role for CNNM4 in mediating transcellular Mg^2+^ transport by basolateral Mg^2+^ extrusion.

## Results

### Generation of CNNM4-knockout mice

**Figure 1:**
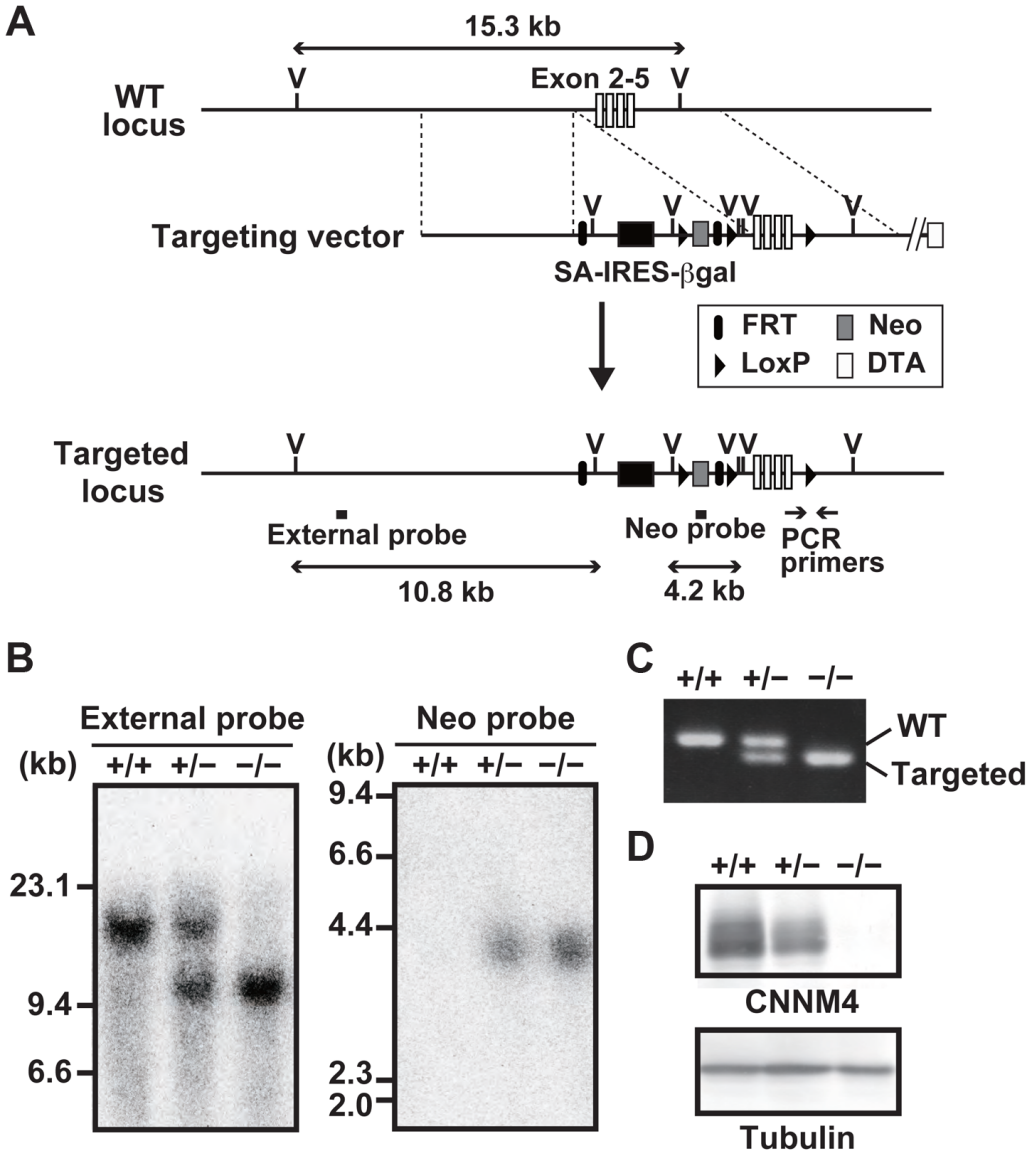
Generation of CNNM4-knockout mice. (A) Targeting strategy. βgal, β-galactosidase gene; DTA, diphtheria toxin A; FRT, Flp recombination target; IRES, internal ribosomal entry site; LoxP, locus of crossing over P1; Neo, neomycin resistance gene; SA, splice acceptor; V, *Eco*RV endonuclease-recognition site. (B) Genomic DNA, isolated from the tails of *CNNM4^+/+^*, *CNNM4^+/−^*, and *CNNM4^−/−^* mice was digested with *Eco*RV and hybridized with the external or neo probes, as schematically shown in (A). (C) PCR was performed using the genomic DNA as a template with the oligonucleotide primers schematically shown in (A). (D) Lysates of the colon were subjected to immunoblotting analyses with the anti-CNNM4 antibody.

To reveal the physiological function of CNNM4, we generated CNNM4-knockout mice. For this purpose, we used a commercially available embryonic stem (ES) cell clone, which possesses the neomycin-resistance gene cassette inserted in the genomic region between the first and second exons of *CNNM4* by homologous recombination (Figure 1A). Chimeric heterozygous mice were obtained by blastocyst injection of the ES cells, and CNNM4-knockout mice were obtained by breeding. Successful recombination in the genomic DNA obtained from *CNNM4^+/−^* and *CNNM4^−/−^* mice was confirmed by Southern blotting (Figure 1B) and routine genotyping was done by PCR (Figure 1C). The gene cassette contains the splice acceptor sequence that forces mRNA splicing to occur artificially at the acceptor sequence, and the resulting mRNA is truncated after the second exon. Indeed, immunoblotting analyses with the anti-CNNM4 antibody (Figure S1) confirmed that *CNNM4^−/−^* mice lack expression of endogenous CNNM4 protein (Figure 1D). Both *CNNM4^+/−^* and *CNNM4^−/−^* mice were viable, with no gross abnormalities.

### Basolateral localization of CNNM4 in the intestinal epithelia

**Figure 2:**
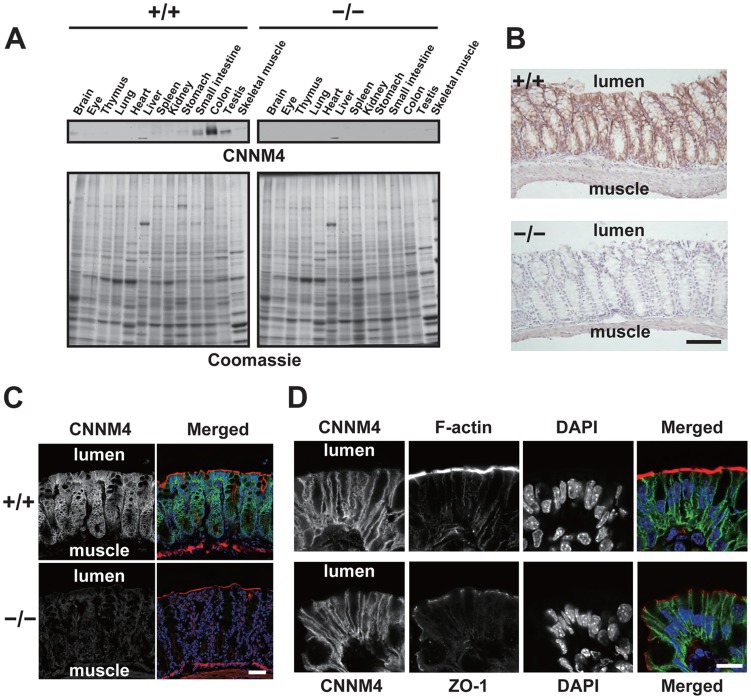
Basolateral localization of CNNM4 in the intestinal epithelia. (A) Lysates of various organs obtained from 2-month-old *CNNM4^+/+^* and *CNNM4^−/−^* mice were subjected to immunoblotting analyses with the anti-CNNM4 antibody. Coomassie-stained images are also indicated. (B) Cryosections of the colon were subjected to immunohistochemical staining with the anti-CNNM4 antibody. Bar, 100 µm. (C) Cryosections of the colon were subjected to immunofluorescence staining with the anti-CNNM4 antibody (green), phalloidin (red), and DAPI (blue). Monochrome images for CNNM4 are also indicated. Bar, 50 µm. (D) Colonic epithelia facing the lumen were subjected to immunofluorescence staining with anti-CNNM4 antibody (green), DAPI (blue), and phalloidin (red, upper panels), or anti-ZO-1 antibody (red, lower panels). Monochrome images for each signal are also indicated. Bar, 10 µm.

Immunoblotting analyses of lysates obtained from various organs showed that CNNM4 is highly expressed in the small intestine and colon (Figure 2A), consistent with the previously reported analyses at mRNA level [18]. We next performed immunohistochemical staining to examine the expression pattern in the colon. As shown in Figure 2B, positive CNNM4 signals were specifically observed at the mucosal epithelial layer, with no significant signals at the muscular layer. Counterstaining of the tissue samples obtained from *CNNM4^−/−^* mice showed no positive signals, thus confirming that the signal at the mucosal epithelia properly reflects the localization of endogenous CNNM4.

To precisely determine the subcellular localization of CNNM4, we also performed immunofluorescence microscopy. Low-magnification images confirmed the specific expression of CNNM4 in the mucosal epithelia (Figure 2C). In the high-magnification images, positive signals for CNNM4 were mostly observed at the plasma membrane, but were clearly separated from those for F-actin, immediately beneath those for ZO-1 (Figure 2D). F-actin staining strongly labels the apical membrane of the intestinal epithelia [19], and ZO-1 is a marker for tight junctions in the colonic mucosa [20], which form a physical border between the apical and the basolateral membranes. Thus, these results imply a basolateral localization of CNNM4 in the colon epithelia.

To further confirm the basolateral localization of CNNM4, we ectopically expressed CNNM4-FLAG in MDCK cells, which maintain a highly polarized epithelial character in culture. As shown in Figure S2, the expressed CNNM4-FLAG proteins co-localized with Na^+^/K^+^ ATPase (basolateral marker), immediately beneath ZO-1.

### Malabsorption of magnesium in CNNM4-knockout mice

**Figure 3:**
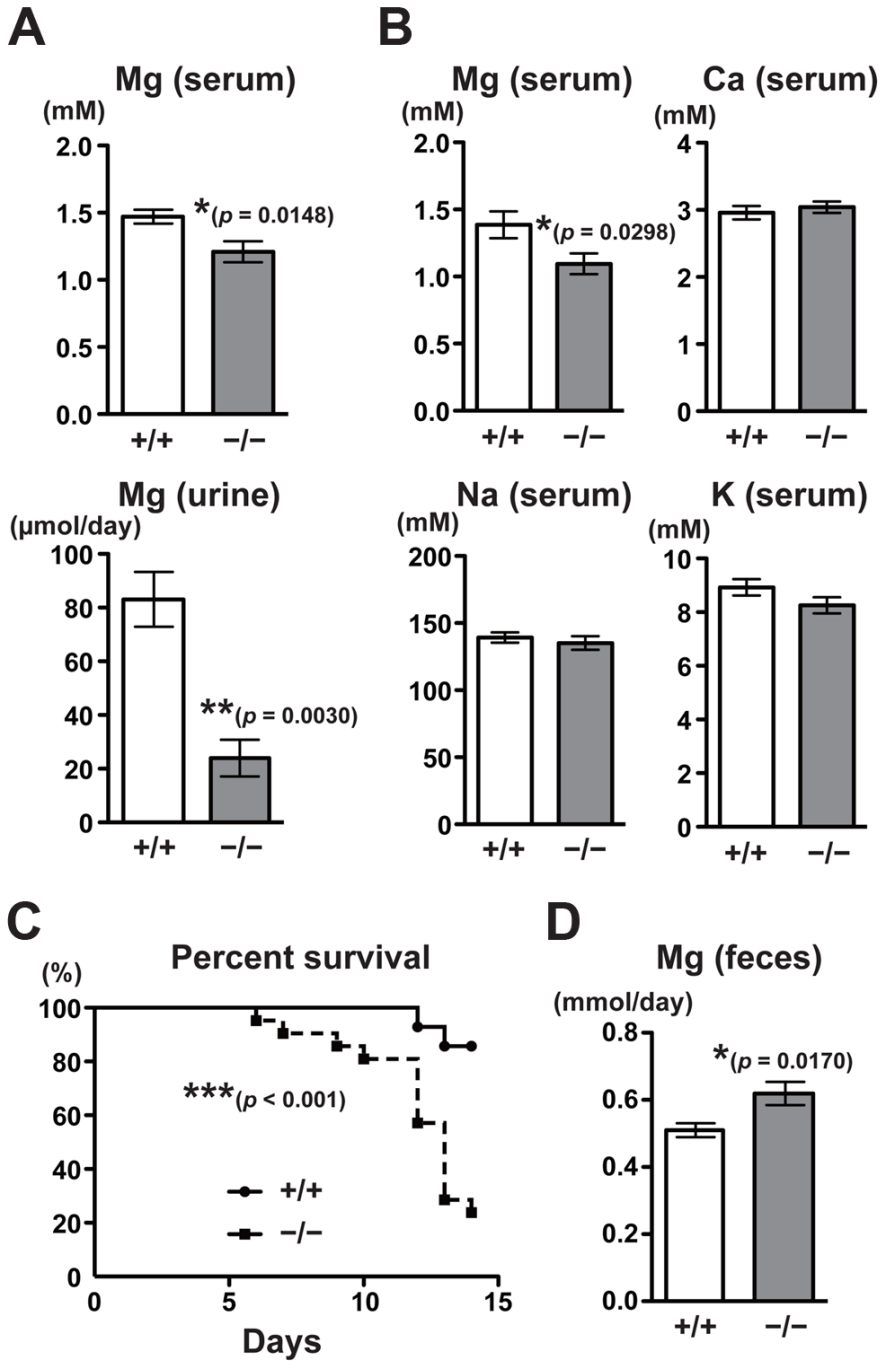
Malabsorption of magnesium in CNNM4-KO mice. (A) Magnesium quantitation in serum (n = 9) and urine (n = 4) obtained from 2-month-old *CNNM4^+/+^* and *CNNM4^−/−^* mice. The data are shown as mean ± s.e.m.. *P*-values were determined by Student's two-tailed *t*-test (unpaired). *p<0.05, **p<0.01. (B) Serum samples were subjected to elemental analyses using ICP-ES. The data are shown as mean ± s.e.m. (n = 14). *P*-values were determined by Student's two-tailed *t*-test (unpaired). *p<0.05. (C) Survival of *CNNM4^+/+^* (n = 11) and *CNNM4^−/−^* (n = 18) mice on a magnesium-deficient diet. ***p<0.001; *p*-values were determined using the log-rank test. (D) Magnesium quantitation in feces. The data are shown as mean ± s.e.m. (n = 8). *P*-values were determined by Student's two-tailed *t*-test (unpaired). *p<0.05.

The fact that CNNM4, a putative Mg^2+^ transporter, localizes to the basolateral membrane of the intestinal epithelia suggests the involvement of CNNM4 in the regulation of magnesium homeostasis. To explore this possibility, we analyzed the magnesium levels in *CNNM4^−/−^* mice maintained on a normal diet (CLEA Rodent Diet CE-2 containing 0.34% magnesium). Magnesium quantitation, using the colorimetric reagent Xylidyl Blue-I, showed that *CNNM4^−/−^* mice had a significantly lower serum magnesium concentration: an approximately 18% decrease was observed in comparison to *CNNM4^+/+^* mice (Figure 3A). Moreover, the magnesium level in urine was drastically reduced, by approximately 71% (Figure 3A). These results demonstrate that *CNNM4^−/−^* mice have altered magnesium regulation. To examine whether this alteration was specific to magnesium, we used inductively coupled plasma-emission spectroscopy (ICP-ES) to examine the levels of several major metal elements in serum. As shown in Figure 3B, the levels of sodium, potassium, and calcium were not affected in *CNNM4^−/−^* mice, whereas the magnesium level was significantly reduced. We then observed mice fed a magnesium-deficient diet (containing 0.0027% magnesium) and found a significant increase in mortality in *CNNM4^−/−^* mice (Figure 3C), indicating that *CNNM4^−/−^* mice have abnormal magnesium homeostasis.

Magnesium homeostasis is regulated by the balance between intestinal absorption and renal excretion. The decrease in renal excretion can be considered to reflect a compensatory response to maintain magnesium levels during hypomagnesemia caused by intestinal malabsorption. To directly measure the effect on intestinal absorption, we analyzed the magnesium content in feces. As shown in Figure 3D, there was significantly higher excretion of magnesium in feces in *CNNM4^−/−^* mice (22% increase compared to *CNNM4^+/+^* mice), without a significant difference in the quantity of food ingested. These symptoms are very similar to those of the *TRPM7*-mutant mice, which have defects in intestinal magnesium absorption [7]. Collectively, these results indicate that CNNM4-deficiency results in malabsorption of magnesium at the intestine.

### Mg^2+^ extrusion by CNNM4

**Figure 4:**
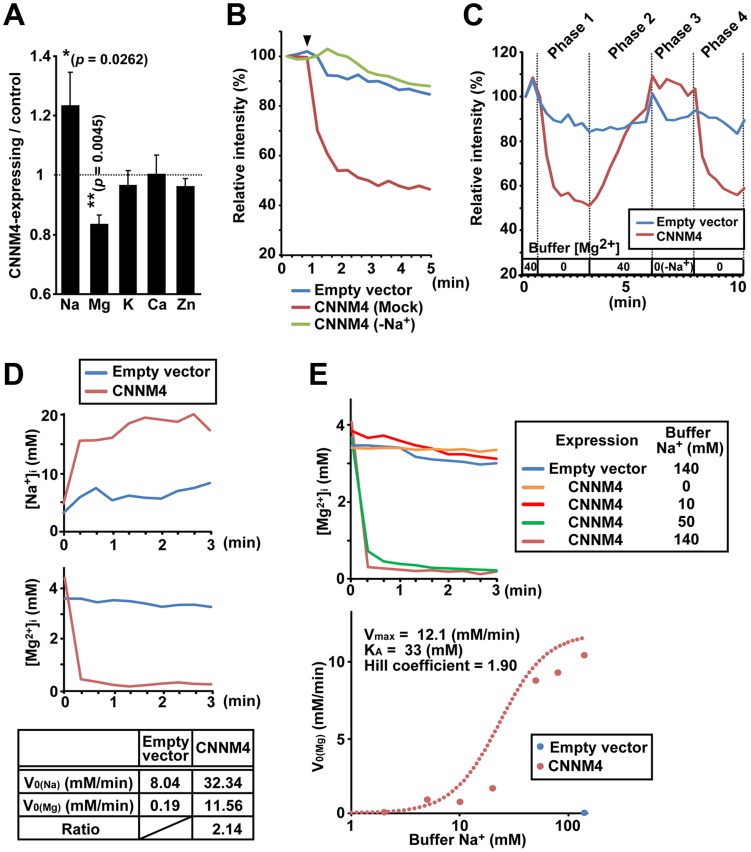
Mg^2+^ extrusion by CNNM4. (A) Lysates of HEK293 cells transfected with CNNM4-FLAG were subjected to ICP-ES analyses. Relative amount of each element (CNNM4-expressing cells/control cells) is shown as mean ± s.e.m. (n = 7). *P*-values were determined by Student's two-tailed t-test (paired). *p<0.05, **p<0.01. (B) HEK293 cells expressing CNNM4-FLAG were loaded with Magnesium Green, and then subjected to Mg^2+^ depletion at the indicated time point (arrowhead). The experiment was repeated in a Na^+^-depleted extracellular solution (−Na^+^) by replacing NaCl with NMDG-Cl. The means of relative fluorescence intensities of 10 cells are indicated. (C) HEK293 cells expressing CNNM4-FLAG were loaded with Magnesium Green and then subjected to time-lapse imaging analyses under various extracellular solutions. The Mg^2+^ concentration in the extracellular solution is indicated (−Na^+^: NaCl in the buffer was replaced with NMDG-Cl). The means of relative fluorescence intensities of 10 cells are indicated. (D) HEK293 cells expressing CNNM4-FLAG were loaded with SBFI or Mag-fura2, and then subjected to Mg^2+^ depletion at 0 min. The data are shown as the means of [Na^+^]_i_ (SBFI-loaded cells, top) and [Mg^2+^]_i_ (Mag-fura2-loaded cells, middle) from 6 independent experiments (10 cells for each experiment). Initial velocities of Na^+^ influx (V_0 (Na)_) and Mg^2+^ efflux (V_0 (Mg)_), and the ratio of CNNM4-dependent Na^+^ influx versus Mg^2+^ efflux are also indicated (bottom). See Materials and Methods for details. (E) HEK293 cells expressing CNNM4-FLAG were loaded with Mag-fura2, and subjected to Mg^2+^ depletion at 0 min with extracellular Mg^2+^-free buffers containing various concentrations of Na^+^. Top: Time course of [Mg^2+^]_i_ (means of 3 independent experiments, and 10 cells for each experiment). Bottom: Values for V_0 (Mg)_ are plotted against Na^+^ concentrations in the buffer. Hill-type curve is also indicated (dotted line).

To clarify the molecular function of CNNM4, we first examined the effect of CNNM4-overexpresion on the intracellular levels of major metal elements by using ICP-ES. As shown in Figure 4A, HEK293 cells transfected with CNNM4-FLAG contained more sodium and less magnesium in comparison to control vector-transfected cells, consistent with the occurrence of Mg^2+^ extrusion. Other analyzed elements (potassium, calcium, and zinc) showed no significant differences. We next performed imaging analyses with Magnesium Green, a fluorescent indicator for Mg^2+^. HEK293 cells transfected with CNNM4-FLAG were first loaded with Mg^2+^ by bathing them in a solution containing 40 mM Mg^2+^, which was then exchanged with a Mg^2+^-free solution to artificially promote Mg^2+^ extrusion. As shown in Figure 4B, the intensity of fluorescent signals in cells expressing CNNM4-FLAG (confirmed by immunofluorescence microscopy, performed after the imaging analyses) rapidly decreased immediately after Mg^2+^ depletion, whereas only a very subtle decrease was observed in empty vector-transfected cells. Thus, CNNM4 is able to stimulate Mg^2+^ extrusion.

The electrical potential across the plasma membrane forces Mg^2+^ to move inward into cells, and thus, energy supply is needed to actively extrude Mg^2+^ to the outside. Many proteins involved in active transport across the plasma membrane utilize the large electrochemical potential of Na^+^. To determine the importance of extracellular Na^+^ in Mg^2+^ extrusion, we first performed Mg^2+^ extrusion assays by replacing Na^+^ in the medium with another cation, N-methyl-D-glucamine (NMDG). In this case, Mg^2+^ extrusion was completely abolished (“−Na^+^” in Figure 4B). We also performed time-lapse imaging analyses for 10 min (Figure 4C and Video S1). Mg^2+^ depletion in the medium caused Mg^2+^ extrusion in CNNM4-expressing cells (Phase 1) and addition of 40 mM Mg^2+^ restored intracellular Mg^2+^ (Phase 2). In the absence of extracellular Na^+^, Mg^2+^ depletion did not induce Mg^2+^ extrusion (Phase 3), but restoration of Na^+^ instantaneously caused Mg^2+^ extrusion (Phase 4). Such tight coupling between the presence of extracellular Na^+^ and the occurrence of Mg^2+^ extrusion further supports the notion that CNNM4 stimulates Na^+^/Mg^2+^ exchange; this is also consistent with the sodium increase observed in CNNM4-expressing cells (Figure 4A).

To determine whether the rapid restoration of intracellular Mg^2+^ in 40 mM Mg^2+^ media is caused by the reverse action of CNNM4, we performed similar time-lapse imaging analyses using cells treated with Cobalt (III) hexammine (CoHex), which broadly inhibits channel-mediated Mg^2+^ influx [21], [22]. CoHex treatment significantly inhibited the Mg^2+^ recovery (Figure S3A), suggesting that some Mg^2+^ channels are involved in the Mg^2+^ recovery process. For more detailed characterization of the Mg^2+^ uptake in CNNM4-expressing cells, we performed a quantitative imaging analyses by using a less-sensitive, but ratiometric fluorescent probe Mag-fura2. Cells were bathed in extracellular solutions containing various concentrations of Mg^2+^ and Na^+^. Unlike 40 mM extracellular Mg^2+^, 10 mM Mg^2+^ was not sufficient to load CNNM4-expressing cells when extracellular Na^+^ was set to 78.1 mM (Figure S3B). However, when extracellular Na^+^ was depleted (0 mM), CNNM4-expressing cells incorporated significant amount of Mg^2+^ even at 10 mM. Furthermore, we observed that even though the Mg^2+^ level in CNNM4-expressing cells was lower than that in the control cells before loading, it became much higher after the loading procedure with 10 mM Mg^2+^, 0 mM Na^+^ solution, and then returned to the basal level when extracellular Mg^2+^ was removed. These data strongly suggest the occurrence of the reverse action of CNNM4 and corroborate our notion that CNNM4 stimulates Na^+^/Mg^2+^ exchange.

### Electroneutral Na^+^/Mg^2+^ exchange by CNNM4

To characterize the molecular function of CNNM4 in more detail, we next performed electrophysiological analyses on CNNM4 expressed in HEK293 cells. As shown in Figure S4A–C, CNNM4 expression induced no significant electronic currents, while CNNM2 expression generated an inward current of Na^+^, as reported previously [12]. To directly measure Mg^2+^ extrusion, we next performed simultaneous Mg^2+^ imaging and electrophysiological recording experiments. The exchange of the extracellular solution with an Mg^2+^-free solution stimulated rapid Mg^2+^ decrease without inducing significant electronic currents in CNNM4-expressing cells (Figure S4D–E). These results suggest the possibility that CNNM4 might exchange 2 Na^+^ and 1 Mg^2+^, and thus, it is electroneutral. Therefore, we performed quantitative imaging analyses of intracellular Na^+^ and Mg^2+^ by using ratiometric fluorescent probes, sodium-binding benzofuran isophthalate (SBFI) and Mag-fura2, respectively. As shown in Figure 4D, Mg^2+^ depletion from the extracellular medium induced not only the decrease of intracellular Mg^2+^ but also the increase of intracellular Na^+^. In addition, the molar ratio of increased Na^+^ and decreased Mg^2+^ was calculated to be 2.14∶1, which is roughly consistent with the electroneutral exchange of Na^+^ and Mg^2+^ (2∶1). To quantitatively assess the dependency of Mg^2+^ extrusion on the presence of extracellular Na^+^, we performed Mg^2+^ extrusion assays by changing the concentration of extracellular Na^+^. Extracellular Na^+^ accelerated Mg^2+^ extrusion in a dose-dependent manner, and the Hill coefficient was calculated to be 1.90, a value close to 2 (Figure 4E). This result suggests that there are 2 or more Na^+^-binding sites in CNNM4, which also agrees with the characteristic of 2 Na^+^/1 Mg^2+^ exchanger.

### Hypomineralization of the tooth enamel in CNNM4-knockout mice

**Figure 5:**
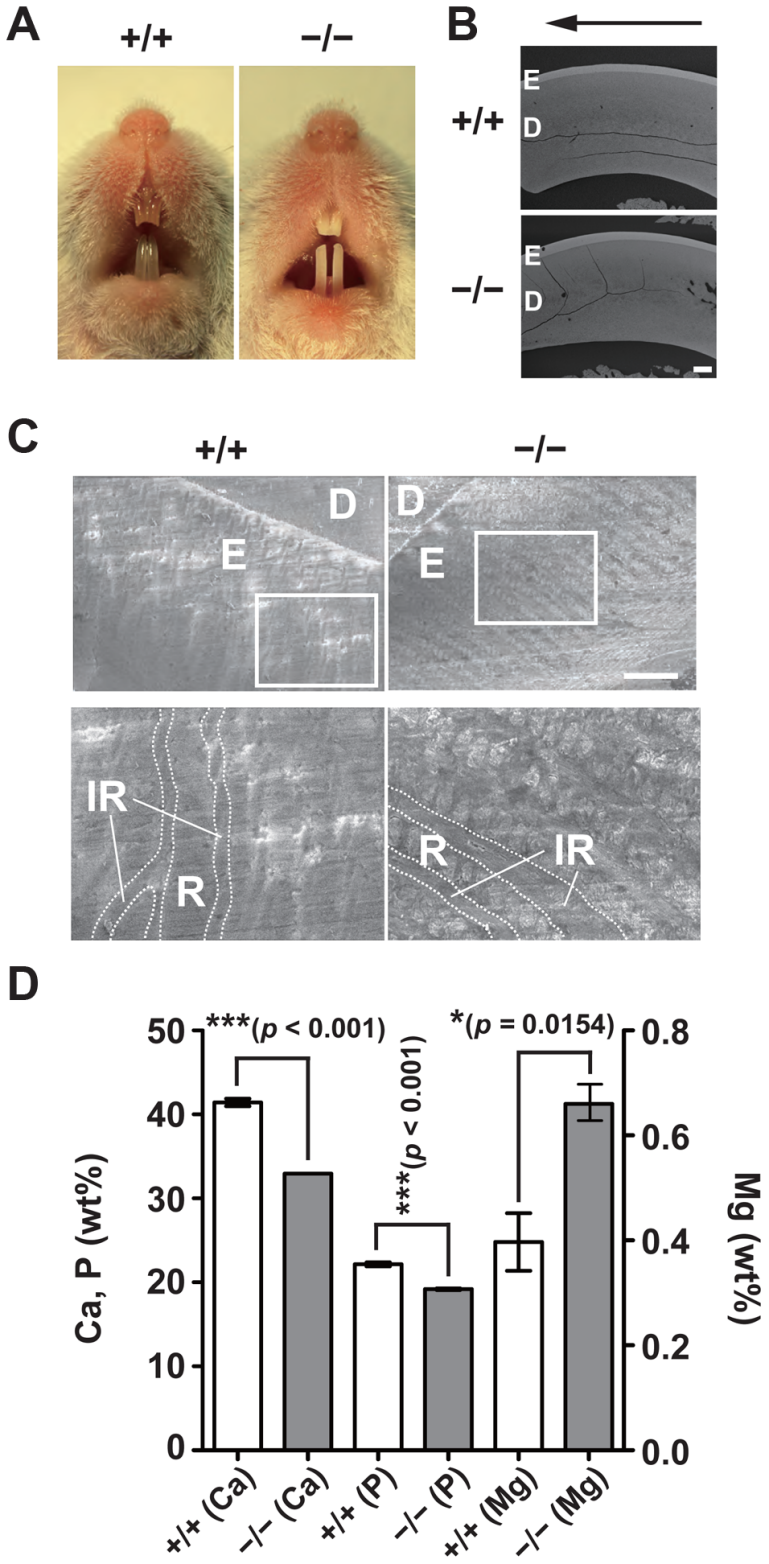
Hypomineralization of the tooth enamel in CNNM4-knockout mice. (A) Oral photographs showing incisors of 2-month-old *CNNM4^+/+^* and *CNNM4^−/−^* mice. (B) Backscattered SEM images showing incisors of 2-month-old *CNNM4^+/+^* and *CNNM4^−/−^* mice. An arrow shows incisal direction. E, enamel; D, dentine. Bar, 200 µm. (C) SEM images showing the mature enamel regions of incisors. Magnified images of the boxed areas are also indicated. E, enamel; D, dentine; R, enamel rod; IR, inter-rod area. Bar, 20 µm. (D) The mineral content of the incisor enamel. The mineral content is expressed as a weight percent (wt%). The data are shown as mean ± s.e.m. (n = 3). *P*-values were determined by Student's two-tailed *t*-test (unpaired). *p<0.05, ***p<0.001.

One of the common features of Jalili syndrome, which is caused by mutations in *CNNM4*, is AI, the malformation of tooth enamel [16], [17]. We noticed that *CNNM4^−/−^* mice displayed abnormal teeth with chalky-white discoloration (Figure 5A), which is typically observed in mice with defective amelogenesis. This phenotype was apparent as early as 3 weeks of age and was observed in all *CNNM4^−/−^* mice examined. To characterize the abnormality in amelogenesis, we subjected maxillary incisors to analyses with scanning electron microscopy (SEM). The low-magnification images showed that the thickness of the enamel layer in *CNNM4^−/−^* mice was not so different from that in *CNNM4^+/+^* mice (Figure 5B). However, the high-magnification images showed that the enamel rods were immature and the inter-rod area was increased in *CNNM4^−/−^* mice (Figure 5C). We then subjected the samples to composition analyses using energy dispersive X-ray spectrometry (EDX). As shown in Figure 5D, the levels of both calcium and phosphorus were significantly decreased in *CNNM4^−/−^* mice, confirming the occurrence of hypomineralization.

### Basolateral localization of CNNM4 in the ameloblasts

**Figure 6:**
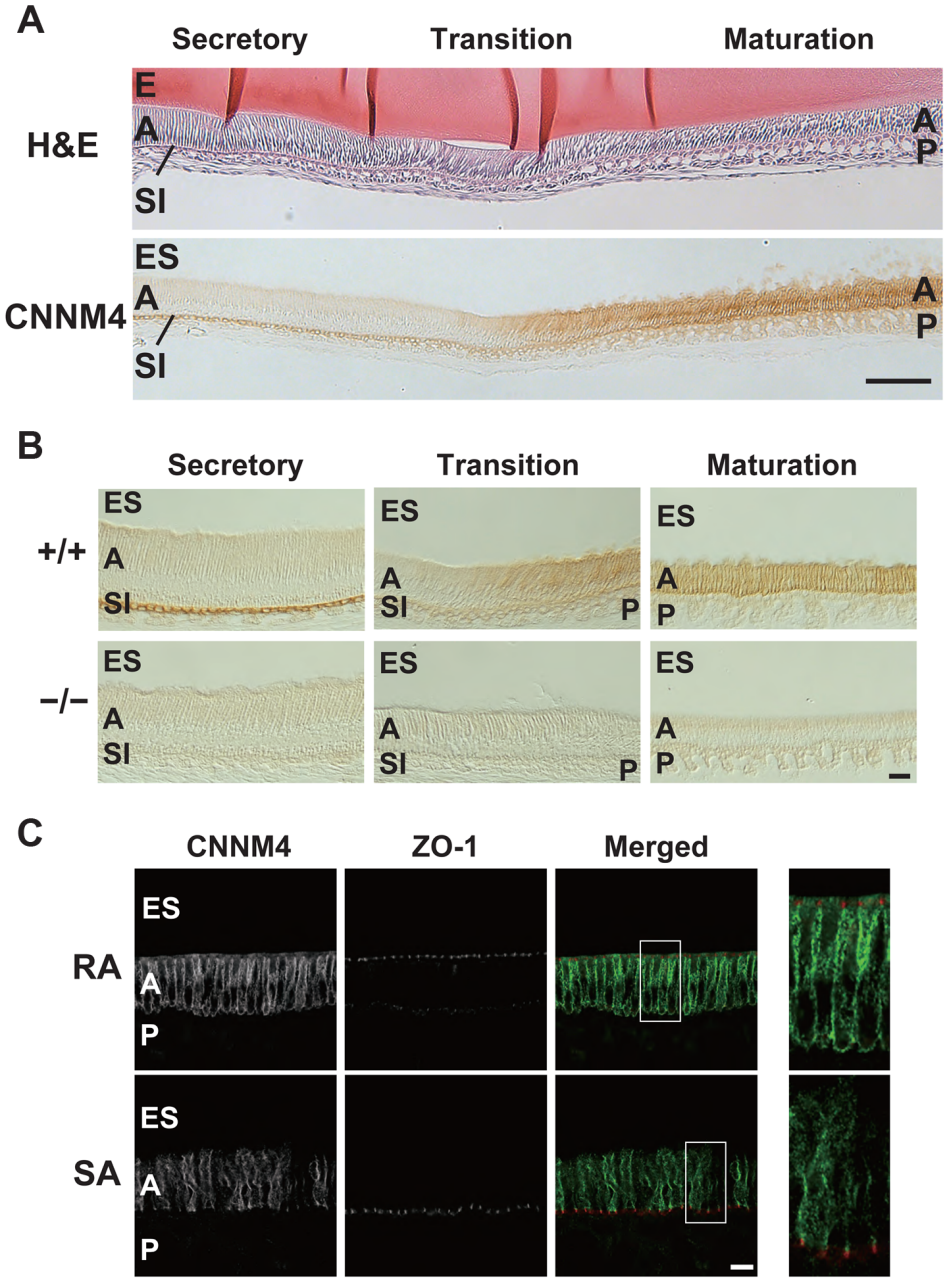
Basolateral localization of CNNM4 in the ameloblasts. (A, B) The enamel-forming tissues of 6-week-old mice were subjected to H&E staining (A) and immunohistochemical staining with the anti-CNNM4 antibody. The specimens were observed with DIC microscope (B). E, enamel; ES, enamel space; A, ameloblast; SI, stratum intermedium; P, papillary layer. Bar, 100 µm (A), 20 µm (B). (C) Cryosections were double-stained with anti-CNNM4 (green) and anti-ZO-1 (red) antibodies, and subjected to immunofluorescence microscopy. RA, ruffle-ended ameloblast; SA, smooth-ended ameloblast; ES, enamel space; A, ameloblast; P, papillary layer. Magnified images of the boxed areas in the merged images are also indicated. Bar, 10 µm.

To explore the role of CNNM4 in amelogenesis, we performed immunohistochemical staining to examine the localization of CNNM4 in the enamel-forming tissue. Enamel formation occurs in the area covered by ectodermally-derived epithelial cells, so-called ameloblasts [23]. The ameloblasts first deposit a complex extracellular matrix composed of enamel proteins (secretory stage), and then come to maturity, with a shortened morphology, and promote mineralization of the enamel (maturation stage). During the secretory stage, positive signals of CNNM4 were observed specifically at the stratum intermedium (SI) layer, but not in the ameloblasts (Figure 6A–B). However, the expression pattern significantly changes at the maturation stage, with strong positive signals in the ameloblasts themselves.

Mature ameloblasts are known to undergo repetitive cycles of transdifferentiation between ruffle-ended (RA) and smooth-ended (SA) ameloblasts, which can be discerned by ZO-1-staining [24]. Immunofluorescence staining showed that CNNM4 exists throughout the basolateral membrane immediately beneath the ZO-1 signals in the RA-type ameloblasts, which possess dot-like accumulations of ZO-1 at the cell-cell contact sites facing the enamel-forming area (Figure 6C). It should be noted that this basolateral localization pattern of CNNM4 in RA-type ameloblasts is quite similar to that observed in intestinal epithelia (Figure 2D), suggesting that CNNM4 promotes Mg^2+^ removal from the maturing enamel. Indeed, the elemental analyses of the mature enamel with EDX indicated that the magnesium levels were significantly increased in *CNNM4^−/−^* mice (Figure 5D).

**Figure 7:**
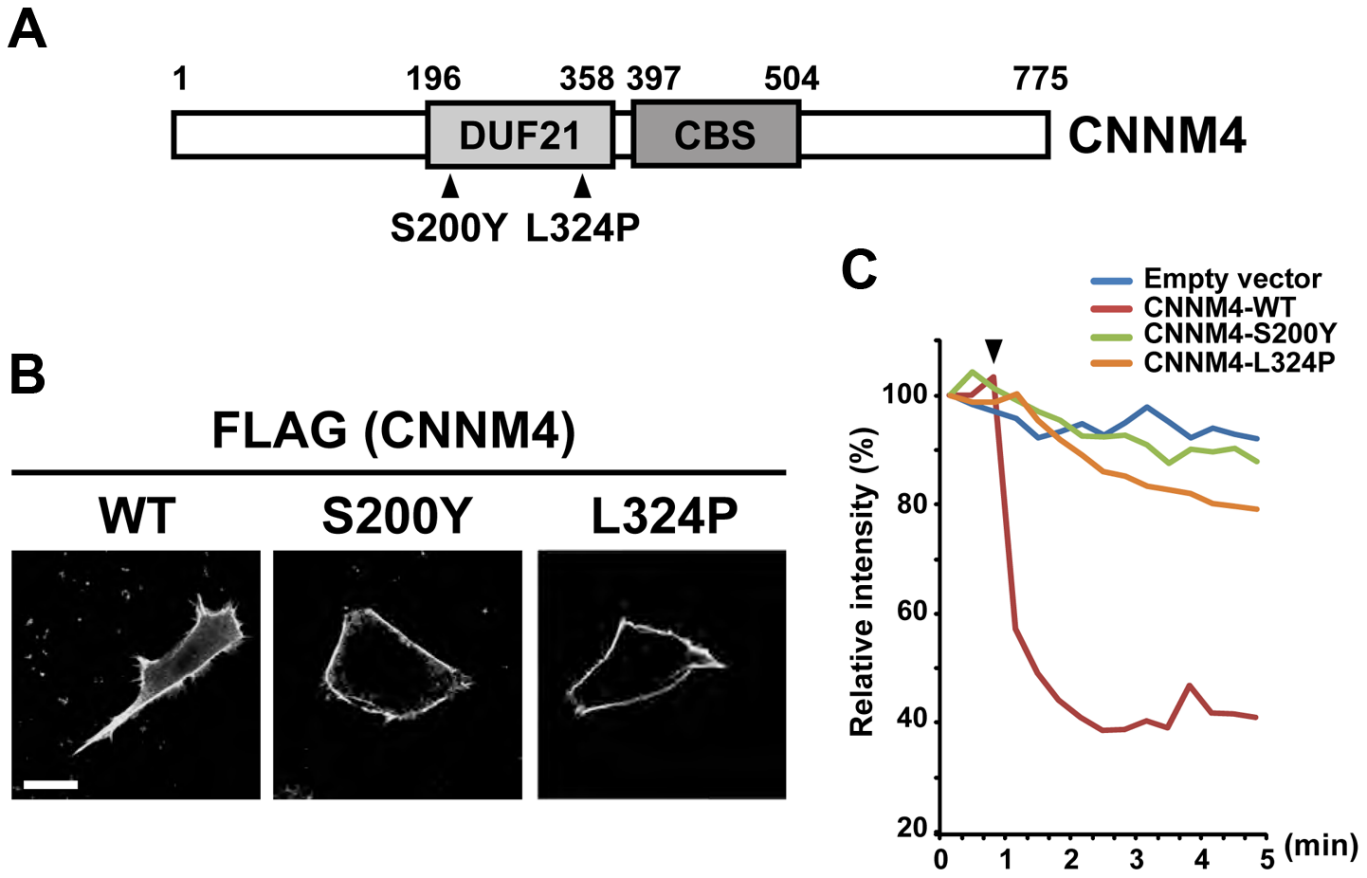
Mutations associated with Jalili syndrome abolish Mg^2+^ extrusion. (A) Schematic illustration of CNNM4 and point mutations found in patients with Jalili syndrome. The evolutionarily conserved DUF21 and CBS domains are boxed and the amino acid residue numbers are indicated. (B) HEK293 cells transfected with the WT and mutant CNNM4-FLAG constructs were subjected to immunofluorescence staining with the anti-FLAG antibody. Bar, 10 µm. (C) HEK293 cells transfected with the WT and mutant CNNM4-FLAG constructs were subjected to Mg^2+^ extrusion assays. The arrowhead indicates the starting point of Mg^2+^ depletion. The means of relative fluorescence intensities of 10 cells are indicated.

To ascertain the functional importance of Mg^2+^ extrusion by CNNM4, we examined whether missense point mutations in *CNNM4*, which have been reported to occur in the patients of Jalili syndrome [16], [17], have any effects on Mg^2+^ extrusion activity. We tested the effect of two different point mutations, viz., S200Y and L324P, both of which occur in the evolutionarily conserved DUF21 domain (Figure 7A). When these mutants were expressed in HEK293 cells, they localized to the plasma membrane, similarly to wild-type (WT) CNNM4 (Figure 7B). However, both mutants showed very weak, if any, Mg^2+^ extrusion activity in comparison to WT CNNM4 (Figure 7C). Therefore, a dysfunction in Mg^2+^ extrusion, caused by mutations in this gene, probably underlies this little understood human disease.

### No symptoms of CRD in CNNM4-knockout mice

Another feature of Jalili syndrome is CRD, which is characterized with the degeneration of rod and cone photoreceptors in the retina [16], [17]. To investigate the integrity of retinal function of *CNNM4^−/−^* mice, we performed histological and electroretinogram (ERG) analyses. To observe the retinal histology, we stained retinal sections from 2-month-old (young adult) *CNNM4^−/−^* mice with toluidine blue. We found that the retinal layers were normal and no symptom of retinal degeneration was observed in the retina of *CNNM4^−/−^* mice (Figure S5A). We also performed immunofluorescent analysis in the *CNNM4^−/−^* retina, using markers of photoreceptor, bipolar, and horizontal cells. Outer segments of rod and cone photoreceptors stained with anti-rhodopsin and cone opsins (M-opsin and S-opsin) are normal in the *CNNM4^−/−^* retina (Figure S5B). Cone photoreceptor synaptic terminals stained with Peanut Agglutinin (PNA) are also localized normally in the outer plexiform layer (OPL). Photoreceptor synaptic ribbons stained with the anti-Ctbp2 antibody showed horseshoe-like structure in the vicinity of dendritic tips of bipolar cells stained with the anti-mGluR6 antibody both in *CNNM4^+/+^* and *CNNM4^−/−^* mice, and dendrites of rod ON-bipolar cells stained with anti-PKC-α antibody and processes of horizontal cells stained with the anti-Calbindin antibody were properly extended into the OPL in the *CNNM4^−/−^*retina. To evaluate the retinal function, we recorded ERGs from *CNNM4^−/−^* mice. As shown in Figure S5C, no obvious difference was observed between 2-month-old *CNNM4^+/+^* and *CNNM4^−/−^* mice in their ERGs under both scotopic and photopic conditions, which reflects the functions of rods and cones, respectively (a-wave in scotopic condition 1.0 log stimuli: +/+, 280±57 µV; −/−, 251±23; unpaired *t*-test: *p* = 0.6204; a-wave in photopic condition 1.0 log stimuli: +/+, 11.3±1.9 µV; −/−, 8.1±0.9; *p* = 0.1966; b-wave in scotopic condition 1.0 log stimuli: +/+, 619±115 µV; −/−, 563±44; *p* = 0.6366; b-wave in photopic condition 1.0 log stimuli: +/+, 163±24 µV; −/−, 126±23; *p* = 0.2937; +/+, n = 5; −/−, n = 6).

Retinal dysfunction occasionally becomes evident with age. Indeed, knockout mice for *RP3*, one of causative genes of human hereditary retinal diseases [25], do not show an apparent loss of the retinal cells at 1 month of age, but degeneration of photoreceptor cells has occurred at 6 months [26]. Therefore, histological analyses of the retina of 6-month-old *CNNM4^−/−^* mice were performed. However, we did not observe any signs of histological abnormalities (Figure S5A–B). We also recorded ERGs from 6-month-old *CNNM4^−/−^* mice and again observed normal ERGs under both scotopic and photopic conditions (Figure S5C).

## Discussion

In this study, we have shown that CNNM4 localizes to the basolateral membrane of epithelial cells and extrudes Mg^2+^. Theoretically, Mg^2+^ extrusion requires an energy supply to overcome the inward-oriented force on Mg^2+^ diffusion imposed by the membrane potential. A Na^+^-coupling Mg^2+^ extrusion mechanism has long been suggested, and indeed, various types of mammalian cells possess Na^+^/Mg^2+^ exchange activity [27], [28]. It was recently reported that SLC41A1 can biochemically function as a Na^+^/Mg^2+^ exchanger when expressed in HEK293 cells [29]. It is expressed ubiquitously [30], and the ectopically expressed SLC41A1 in MDCK cells localizes at the basolateral membrane [31]. Therefore, SLC41A1 may also be involved in the regulation of directional Mg^2+^ transport across the intestinal epithelia. However, it should be noted that the speed of Mg^2+^ extrusion by CNNM4 (reaching plateau after 1∼2 min) is much faster than that by SLC41A1 (after ∼10 min) [29]. Such a rapid Mg^2+^ extrusion has not been reported in the previous studies characterizing the endogenous Mg^2+^ extrusion systems in non-intestinal cells [27], [28]. Thus, CNNM4 appears to be a qualitatively different, high capacity type of Mg^2+^ extrusion molecule, which may have a specialized role in the intestinal epithelia. Magnesium absorption from the intestine is essential for magnesium homeostasis, and 100–150 mg magnesium is daily absorbed from the intestine in humans [32]. To absorb such a large amount of magnesium through the intestinal epithelia, the magnesium transport system in the intestine should be highly active. It is known that both paracellular and transcellular pathways are functional and play important roles in the intestinal magnesium absorption [33]. In the transcellular pathway, Mg^2+^ entry into the intestinal epithelial cells is mediated by apically localized Mg^2+^-permeable channels TRPM6/7 that can rapidly incorporate Mg^2+^ [6], [7]. Therefore, it is very reasonable that Mg^2+^ extrusion from the basolateral membrane is mediated by high capacity transporters, such as CNNM4, to achieve efficient transcellular Mg^2+^ transport through intestinal epithelia.

*CNNM4^−/−^* mice showed a defect in magnesium absorption, but were viable, without any significant observable phenotype when fed a normal diet. CNNM proteins comprise a family of 4 related proteins, CNNM1–4 [10], and thus, the mild phenotype of *CNNM4^−/−^* mice can be ascribed to the functional complementation by other CNNM family proteins. CNNM4 is expressed in the intestine, but not in the kidney, and thus, it will not affect renal reabsorption, the other key process in the regulation of magnesium homeostasis. The amount of magnesium reabsorbed from the glomerular filtrate is estimated to be about 10 times that absorbed from digested food. Therefore, the absence of CNNM4 in the kidney raises the next important question of what molecule is responsible for Mg^2+^ extrusion from distal convoluted tubule (DCT) cells in the kidney, where TRPM6 is expressed at the apical membrane and where transcellular Mg^2+^ transport occurs [34]. Two previous papers have reported strong expression and localization of CNNM2 at the basolateral membrane of the DCT cells [12], [18]. Therefore, it can be assumed that CNNM2 plays an important role in renal reabsorption of magnesium at the DCT by mediating transcellular Mg^2+^ transport cooperatively with TRPM6. It should be noted here that SLC41A1 is also expressed in the DCT cells and its gene mutation causes nephronophthisis-related disorder [31]. Because the affected patients did not exhibit any abnormalities in serum or urine magnesium level, the authors speculated that the disease phenotype might result from perturbed intracellular magnesium homeostasis [31]. Future studies using gene knockout mice and detailed analyses of the biochemical properties of these molecules, CNNM2 and SLC41A1, will grant more insight into the individual roles in renal magnesium control.

*CNNM4* is mutated in Jalili syndrome, which is characterized by recessive AI and CRD [16], [17]. Our *CNNM4^−/−^* mice showed no signs of abnormalities in the retinal tissue architecture and function (Figure S5). In contrast, we observed a clear amelogenesis-defective phenotype. In the enamel-forming tissue, CNNM4 is strongly expressed at the basolateral membrane in RA-type ameloblasts. Such a basolateral localization is similar to that observed in the intestinal epithelia and suggests that CNNM4 is involved in the vectorial transport of Mg^2+^ from the enamel-forming areas through the ameloblasts. Indeed, RA-type ameloblasts have tight junctions in the region adjacent to the enamel-forming areas, and form a niche in which active ion transport occurs [24]. The precise role of Mg^2+^ in the enamel-forming process remains unknown, but the striking expression of CNNM4 in RA-type mature ameloblasts suggests that Mg^2+^ needs to be removed from the enamel tissue to promote mineralization of enamel. Indeed, it has been reported that the magnesium content of the enamel is inversely correlated with the extent of mineralization [35]. Further characterization of CNNM4-knockout mice will contribute to a better understanding of this intriguing process in which the most solid tissue in the body is generated.

## Materials and Methods

### Ethics statement

We appropriately treated mice to ameliorate suffering, according to the guidelines for proper conduct of animal experiments (issued by the Science Council of Japan), and received approval for this study from the institutional review board of Osaka University.

### Generation of CNNM4-knockout mice

We purchased an ES clone (ID: EPD0426_1_C08) from EUCOMM, in which the neomycin-resistant gene cassette had been inserted in the genomic region between the first and second exons of *CNNM4* by homologous recombination. The ES cells were used to generate germline chimeras that were bred with C57BL/6J females to generate CNNM4-knockout mice. Southern blot analyses were performed to confirm appropriate recombination. Genomic DNA of mice was digested with *Eco*RV and hybridized with the external or neo probes. Genotyping PCR was performed using the following primer set: 5′-TAACTGTTGGAAGGCTGAGG-3′ and 5′-AGGCAGGGGCTCCCTTTCAT-3′. Mice were maintained under standard specific pathogen-free conditions.

### cDNA and antibody

Human *CNNM4* cDNA was purchased from Invitrogen (IMAGE: 30340626). Amino acid substituted mutants S200Y and L324P were generated with the QuickChange Site-Directed Mutagenesis Kit (Agilent). An anti-CNNM4 rabbit polyclonal antibody was raised in rabbits immunized with bacterially expressed His-CNNM4 proteins (amino acids 546–775) and purified with corresponding GST-tagged recombinant proteins. Anti-ZO-1 mouse monoclonal antibody was generated in the previous study [36] and provided by Dr. Masahiko Itoh (Dokkyo Medical University) and Dr. Mikio Furuse (Kobe University). Anti-mGluR6 guinea pig polyclonal antibody was described previously [37]. Anti-Na^+^/K^+^ ATPase mouse monoclonal antibody (#05-369) and anti-M-opsin rabbit polyclonal antibody (AB5405) were purchased from Merck Millipore. Anti-FLAG rabbit polyclonal antibody (F7425) and anti-PKCα rabbit polyclonal antibody (P4334) were purchased from Sigma-Aldrich. Anti-Ctbp2 mouse monoclonal antibody (612044) was purchased from BD Biosciences. Anti-Rhodopsin (LB-5597) and anti-Calbindin (PC253L) rabbit polyclonal antibodies were purchased from LSL and Calbiochem, respectively. Anti-S-opsin goat polyclonal antibody (sc-14363) was purchased from Santa Cruz Biotechnology. Alexa Fluor 488-conjugated anti-rabbit IgG was purchased from Invitrogen and Sigma-Aldrich. Alexa Fluor 488-conjugated anti-mouse IgG was purchased from Sigma-Aldrich. Alexa Fluor 568-conjugated anti-mouse IgG, and rhodamine-labelled phalloidin were purchased from Invitrogen. Cy3-conjugated anti-rabbit, -goat and -guinea pig IgGs were purchased from Jackson ImmunoResearch Laboratories. Rhodamine-labeled PNA (RL1072) was purchased from Vector Laboratories.

### Expression and RNAi knockdown in culture cells

HEK293 cells and MDCK cells were cultured in Dulbecco's modified Eagle's medium supplemented with 10% fetal bovine serum and antibiotics. Transient expression and knockdown were achieved using LipofectAmine2000 (Invitrogen) to transfect cells with plasmids or siRNAs according to the manufacturer's instruction. Plasmid constructs in the pCMV-Tag 4 vector (Agilent Technologies) were used for expression of CNNM4. For knockdown experiments, duplex siRNAs against human *CNNM4* (Invitrogen), which target the following sequence: CNNM4-siRNA, 5′-GCGAGAGCAUGAAGCUGUAUGCACU-3′, were used. As control, we used siRNA representing a scrambled sequence of CNNM4-siRNA, 5′-GCGACGAAAGUGUCGGUAUCGAACU-3′.

### Immunohistochemistry

For intestine preparation, intestines were dissected from 2-month-old mice, embedded in OCT compound (Sakura Finetechnical), frozen in liquid nitrogen, and then sectioned into at 10-µm sections using a cryostat (Leica). The sections were mounted on glass slides, air-dried, and fixed with phosphate-buffered saline (PBS) containing 4% paraformaldehyde (PFA) for 10 min at 4°C. For mandible preparation, 6-week-old mice were anesthetized and fixed by perfusion with PBS containing 4% PFA. Mandibles were dissected out, fixed with PBS containing 4% PFA for 12 h at 4°C, decalcified with 10% EDTA for 2 weeks, dehydrated with xylene through a graded ethanol series, and embedded in paraffin. Sections (4-µm thick) were cut using a microtome (Leica), and then mounted on glass slides. Slides were heat-treated in Pascal, a pressure chamber (Dako) and cooled at room temperature after deparaffinization and rehydration. Both frozen and paraffin-embedded sections were then incubated with PBS containing 0.3% H_2_O_2_. After blocking with PBS containing 3% fetal bovine serum and 10% bovine serum albumin for 1 h at room temperature, specimens were incubated with the primary antibodies overnight at 4°C, followed by incubation with the peroxidase-conjugated secondary antibodies for 1 h at room temperature. Immunostaining was developed with diaminobenzidine and counterstained with Mayer's haematoxylin. The specimens were observed under a microscope (BX41 equipped with a DP20 camera; Olympus). Differential Interference Contrast (DIC) images were collected using an inverted microscope (IX71 equipped with a DP20 camera; Olympus).

### Immunofluorescence microscopy

Cells cultured on coverglasses were washed with PBS and fixed with 1% formaldehyde for 15 min at room temperature. When stained for ZO-1, cells were permeabilized with 0.5% TritonX-100 in PBS for 10 min at room temperature. When stained for Na^+^/K^+^-ATPase, cells were permeabilized with 0.1% TritonX-100 for 5 min at room temperature. After blocking with PBS containing 3% fetal bovine serum and 10% bovine serum albumin (blocking buffer) for 1 h, cells were incubated for 12 h with the primary antibody diluted in blocking buffer. After 3 washes with PBS, cells were incubated for 30 min with the appropriate secondary antibodies diluted in blocking buffer. Cryosections of intestines and paraffin-embedded sections were prepared as described above. When stained for ZO-1, sections were permeabilized with ice-cold acetone for 3 min after fixation. Fixed sections were blocked and incubated with the primary and secondary antibodies as for cultured cells. After washing with PBS, coverglasses were mounted with Aqueous Mounting Medium PermaFluor (Thermo SCIENTIFIC) and observed with a confocal scanning laser microscope (FLUOVIEW FV1000; Olympus). The procedure of immunofluorescent analysis of retinas was described previously [38], [39]. Mouse eyes were fixed with PBS containing 4% PFA for 30 min or 5 min, embedded in OCT compound, frozen, and sectioned. Frozen 20 µm sections were blocked with PBS containing 5% normal goat serum and 0.5% Triton X-100 for 30 min, and then incubated with primary antibodies for 4 h at room temperature. Slides were washed with PBS three times for 5 min each time and incubated with secondary antibodies for 2 h at room temperature. The specimens were observed with a confocal scanning laser microscope (LSM510; Carl Zeiss).

### ERG recordings

ERG responses were measured after overnight dark adaptation using PuREC system with LED electrodes (Mayo Corporation) [40]. 2- and 6-month-old mice were anesthetized with an intraperitoneal injection of ketamine and xylazine. The mice were stimulated with stroboscopic stimuli of 1.0 log cd-s/m^2^ (photopic units) maximum intensity. 4 levels of stimulus intensities ranging from −4.0 to 1.0 log cd-s/m^2^ were used for the scotopic ERG recordings, and 4 levels of stimuli ranging from −0.5 to 1.0 log cd-s/m^2^ were used for the photopic ERGs. Animals were light adapted for 10 min before the photopic ERG recordings. 8 and 16 responses were averaged for photopic (−4.0 and −3.0 log) and all scotopic recordings, respectively.

### Colorimetric quantitation of magnesium

Mice were fed either a normal diet containing 0.34% magnesium (CLEA Rodent Diet CE-2, CLEA Japan) or a magnesium-deficient diet containing 0.0027% magnesium (CLEA Japan). Blood samples were obtained from 8-week-old mice. These were incubated at 4°C overnight, and serum was then collected by centrifugation at 1,000× *g* for 20 min at 4°C. Urine and feces samples were collected from 2-month-old mice by using metabolic cages (CLEA Japan). Feces were air-dried, incubated with 1 N nitric acid (1∶10; wt∶volume) overnight, and then centrifuged. The magnesium concentration of the supernatant was determined using Xylidyl Blue-I (Wako) according to the manufacturer's instructions.

### ICP-ES

Serum samples were mixed with HCl at a final concentration of 1% and incubated at 95°C for 2 h. Samples were then subjected to elementary analysis with ICPS-8100 (Shimadzu), according to the manufacturer's instructions. The mean of triplicate measurements was used to represent the result of a single sample. The results were normalized to total protein levels, which were determined by the Bradford method.

### Mg^2+^-imaging analyses with Magnesium Green

Mg^2+^-imaging analyses with Magnesium Green were performed as follows. HEK293 cells were incubated with Mg^2+^-loading buffer (78.1 mM NaCl, 5.4 mM KCl, 1.8 mM CaCl_2_, 40 mM MgCl_2_, 5.5 mM glucose, 5.5 mM HEPES-KOH, pH 7.4), including 2 µM Magnesium Green-AM (Invitrogen), for 45 min at 37°C. The cells were rinsed once with loading buffer and viewed using a microscope (IX81 equipped with a DP30BW camera and a USH-1030L mercury lamp; Olympus). Fluorescence was measured every 20 sec (excitation at 470–490 nm and emission at 505–545 nm) under the control of the Metamorph software (Molecular Devices). Then, the buffer was changed to −Mg^2+^ buffer (MgCl_2_ in the loading buffer was replaced with 60 mM NaCl), or to −Mg^2+^−Na^+^ buffer (NaCl in −Mg^2+^ buffer was replaced with NMDG-Cl). The data are presented as line plots (mean of 10 cells). After imaging analyses, cells were fixed with PBS containing 3.7% formaldehyde and subjected to immunofluorescence microscopy to confirm protein expression. Cobalt (III) hexammine was purchased from SIGMA.

### Electrophysiological recordings

pIRES-HcRed plasmids [41] for expressing CNNM2 or CNNM4 were transfected into HEK293 cells with FuGENE6 (Roche). After 24 h, cells were plated on glass coverslips coated with poly-L-lysine (SIGMA) and maintained in normal culture media plus 40 mM MgCl_2_ until use. Patch-clamp experiments under the whole-cell configuration were performed according to Stuiver et al., [12] with minor modifications. The experiments were performed with Axopatch 200B amplifier and Clampex 9.2 data acquisition system (Molecular Devices), and borosilicate patch pipettes had resistances of 5–10 MΩ after filled with the intracellular solution. Voltage steps (1 sec in duration) from the holding potential of 0 mV to potentials between −120 to +70 mV with 10 mV increment were delivered every 4 sec. The density current was obtained from the peak current at −110 mV and was normalized with the membrane capacitance of the cell. The extracellular solutions was 80 mM Na-gluconate, 0 or 20 mM MgSO_4_, 10 mM HEPES (pH 7.35 adjusted with Tris). The intracellular solution was 120 mM NMDG, 120 mM 2-(N-morpholino)-ethanesulfonic acid hydrate, 2 mM MgSO_4_, 10 mM HEPES (pH 7.2 adjusted with H_2_SO_4_). All solutions were adjusted to 295–305 mOsm with sucrose.

Simultaneous Mg^2+^-imaging and electrophysiological recording experiments were performed with IX71 microscope (Olympus) equipped with iXon EM-CCD camera (Andor Technology) and a xenon lamp in Lambda DG-4 illumination system (Sutter Instrument). Borosilicate patch pipettes had resistances of 3–5 MΩ after filled with the intracellular solution containing 2 µM Magnesium Green (non-AM form, Invitrogen). Cells were voltage clamped to −10 mV, and the imaging was started after the fluorescent intensities from the cell became stabilized (20–35 min after the establishment of the whole-cell configuration). The fluorescence was measured every 20 sec (excitation at 470–490 nm and emission at 505–545 nm). Mg^2+^-loading buffer and −Mg^2+^ buffer were used as extracellular solutions. The intracellular solution was 2 mM MgCl_2_, 2 mM NaCl, 5 mM EGTA, 140 mM KCl, 5 mM HEPES (pH 7.25 adjusted with KOH). All solutions were adjusted to 295–305 mOsm with sucrose.

### Ratiometric imaging of Mg^2+^ and Na^+^

HEK293 cells were transfected with expression plasmids for CNNM4, and maintained in normal culture media plus 40 mM MgCl_2_ until use. Mg^2+^ extrusion assays were performed with the abovementioned protocol, with following modifications. Cells were loaded with 2 µM Mag-fura2-AM or 3 µM SBFI-AM (Invitrogen) and viewed using the IX81 microscope (Olympus) equipped with ORCA-Flash 4.0 CMOS camera (Hamamatsu Photonics) and USH-1030L mercury lamp (Olympus). The fluorescence was measured every 20 sec (excitation at 330–350 nm and 370–390 nm, and emission at 505–545 nm), and −Mg^2+^ buffer with various Na^+^ concentrations (prepared by replacing NaCl with NMDG-Cl) was used to stimulate Mg^2+^ efflux.

Intracellular concentrations of free Mg^2+^ and Na^+^ ([Mg^2+^]_i_ and [Na^+^]_i_, respectively) were determined from the following equation:
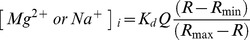
R: the ratio of the signal intensity with 330–350 nm excitation (F_1_) to that with 370–390 nm excitation (F_2_) (R = F_1_/F_2_). R_max_: the maximum value of R. R_min_: the minimum value of R. Q: the ratio of the signal intensity with 370–390 nm excitation under minimum Mg^2+^ or Na^+^ concentration to the signal intensity with 370–390 nm excitation under maximum Mg^2+^ or Na^+^ concentration (F_2min_/F_2max_). K_d_: 1.5 mM for Mag-fura2 [42] and 11.3 mM for SBFI [43], respectively. R_min_, R_max_, F_min_, F_max_ were obtained after each experiment. For Mag-fura2, R_min_, F_min_ were recorded by addition of 6 µM 4-Bromo-A23187 (Wako) and 10 mM EDTA, and R_max_, F_max_ were recorded by incubating the cells under −Mg^2+^ buffer plus 6 µM 4-Bromo-A23187 and 50 mM MgCl_2_. For SBFI, R_max_, F_max_ were recorded by incubating the cells under −Mg^2+^ buffer supplemented with 5 µM Gramicidin (Wako), and R_min_, F_min_ were recorded by incubating the cells under the Na^+^-depleted buffer (a −Mg^2+^ buffer which NaCl is replaced with KCl) with 5 µM Gramicidin. The cells were fixed with PBS containing 3.7% formaldehyde after fluorescence measurement and subjected to immunofluorescence microscopy to confirm protein expression. Difference of [Na^+^]_i_ and [Mg^2+^]_i_ just after Mg^2+^ depletion (between time = 0 and 20 sec) was used to determine the initial velocity of Na^+^ influx (V_0 (Na)_) and Mg^2+^ efflux (V_0 (Mg)_), respectively. The ratio of CNNM4-dependent Na^+^ influx versus Mg^2+^ efflux was calculated as follows:
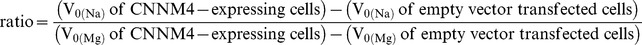
V_max_, K_A_, and Hill coefficient were determined by SigrafW software [44].

### Mg^2+^ loading assays

HEK293 cells were transfected with expression plasmids for CNNM4, and maintained in normal culture media until use. Mg^2+^ loading assays were performed with the abovementioned protocol for ratiometric imaging, with following modifications. Cells were incubated in −Mg^2+^ buffer with 2 µM Mag-fura2-AM for 10 min, 37°C. The cells were once rinsed with −Mg^2+^ buffer and viewed using the same apparatuses. Then, the extracellular solution was changed to buffers with various Mg^2+^ and Na^+^ concentrations (buffer with low Na^+^ concentrations were prepared by replacing NaCl with NMDG-Cl) and incubated for 4 min to load Mg^2+^. Finally, the extracellular solution was changed to −Mg^2+^ buffer to stimulate Mg^2+^ efflux.

### SEM analyses

Maxillae dissected from 2-month-old mice were fixed with 70% ethanol for 5 days, dehydrated in ascending alcohol series, and embedded in methyl methacrylate. After embedding, cutting specimen, and surface polishing, the samples were then coated with room-temperature ionic liquid (1-butyl-3-methylimidazolium tetrafluoroborate), which work as an electric conductor and enables the observation of biological specimen by an SEM [45]. Samples were mounted with carbon adhesion tape on a specimen holder for SEM. Backscattered and secondary electron images were obtained with SEM (VE-9800: Keyence). The composition changes were analyzed with EDX (VE9800: EDAX) attached to the SEM at an accelerating voltage of 8 keV.

## Supporting Information

Figure S1 Characterization of the anti-CNNM4 antibody. Lysates of HEK293 cells transfected with CNNM4-FLAG or CNNM4-siRNA were subjected to immunoblotting analyses with the anti-CNNM4 antibody. The endogenous CNNM4 signal in HEK293 cells is indicated with an arrowhead.
(PDF)

Figure S2 Basolateral localization of ectopically expressed CNNM4 in MDCK epithelial cells. MDCK cells transfected with CNNM4-FLAG were subjected to immunofluorescence staining with the antibodies for FLAG (green) and ZO-1 (red) or Na+/K+ ATPase (red). Horizontal section images (X–Y), at the level of the apical and lateral membranes, and vertically reconstituted images (X–Z) are shown. Vertical sections were taken at the white lines indicated in the X–Y images. Bar, 10 µm.
(PDF)

Figure S3 Characterization of Mg^2+^ influx in CNNM4-expressing cells. (A) HEK293 cells expressing CNNM4-FLAG were loaded with Magnesium Green and then subjected to time-lapse imaging analyses in the presence of 1 or 3 mM CoHex, under various extracellular solutions. The Mg^2+^ concentration in the extracellular solution is indicated. The means of relative fluorescence intensities of 10 cells are indicated. (B) HEK293 cells expressing CNNM4-FLAG were loaded with Mag-fura2, and then subjected to Mg^2+^ loading assays. The cells were incubated with extracellular solutions containing various concentrations of Mg^2+^ and Na^+^ during the indicated period (“Mg^2+^ loading”). The Mg^2+^ and Na^+^ concentrations in the extracellular solutions during the loading period, and the means of [Mg^2+^]_i_ of 4 independent experiments (10 cells for each experiment) are indicated. See Materials and Methods for details.
(PDF)

Figure S4 Electrophysiological recordings. (A) HEK293 cells transfected with the indicated expression constructs were subjected to electrophysiological recordings under whole-cell configuration. The cells were voltage-clamped between −120 mV and 70 mV in steps of 10 mV, and the representative traces of each cell are indicated. (B) Average I–V relationship of control (green), CNNM2- (blue), or CNNM4- (red) expressing cells recorded either in the presence (dotted lines) or absence (solid lines) of extracellular Mg^2+^ (n = 5–6). (C) Current densities at −110 mV recorded either in the presence or absence of extracellular Mg^2+^. Data are presented as mean ± s.e.m. of 5–6 cells. *p* value was determined by Student's two-tailed t-test. n.s.: not significant. (D) HEK293 cells transfected with the indicated constructs were subjected to simultaneous Mg^2+^ imaging and electrophysiological recording experiments. The extracellular solution was changed from Mg^2+^-containing to an Mg^2+^-free solution at the time point indicated by arrowheads. Means of relative fluorescence intensities and current densities (at −10 mV) of 4–6 cells are indicated. (E) Decreased fluorescence intensities and induced current densities were observed during the period between the arrows in (D). Data are presented as mean ± s.e.m. of 4–6 cells. *p* values were determined by Student's two-tailed t-test. ***p<0.001, n.s.: not significant.
(PDF)

Figure S5 Histological and ERG analysis of the retina. (A) Retinal sections from 2- and 6-month old *CNNM4^+/+^* and *CNNM4^−/−^* mice were stained with toluidine blue. Bar, 100 µm. (B) Retinal sections were stained with the indicated antibodies or lectins. Bars, 100 µm (Rhodopsin/PNA; M-opsin/S-opsin); 50 µm (Calbindin/PNA); 10 µm (Ctbp2/mGluR6; Ctbp2/PKCα). (C) Representative ERG waveforms recorded from 2- and 6-month old *CNNM4^+/+^* and *CNNM4^−/−^* mice. Scotopic and photopic ERGs with stroboscopic stimuli of 1.0 log cd-s/m^2^ are shown. GCL, ganglion cell layer; INL, inner nuclear layer; ONL, outer nuclear layer; OPL, outer plexiform layer; OS, outer segment; RPE, retinal pigment epithelium.
(PDF)

Video S1 Time-lapse Mg^2+^-imaging analyses. HEK293 cells expressing CNNM4-FLAG (labeled with asterisks) were loaded with Magnesium Green and then subjected to time-lapse imaging analyses by changing the extracellular solution from phase 1 to phase 4 (see the text for detail).
(MOV)
