# Supplementary figures and images for: Basolateral Mg2+ Extrusion via CNNM4 Mediates Transcellular Mg2+ Transport across Epithelia: A Mouse Model
Source: PLoS Genet. 2013 Dec 5;9(12):e1003983. doi: 10.1371/journal.pgen.1003983 (PMC3854942; doi:10.1371/journal.pgen.1003983)

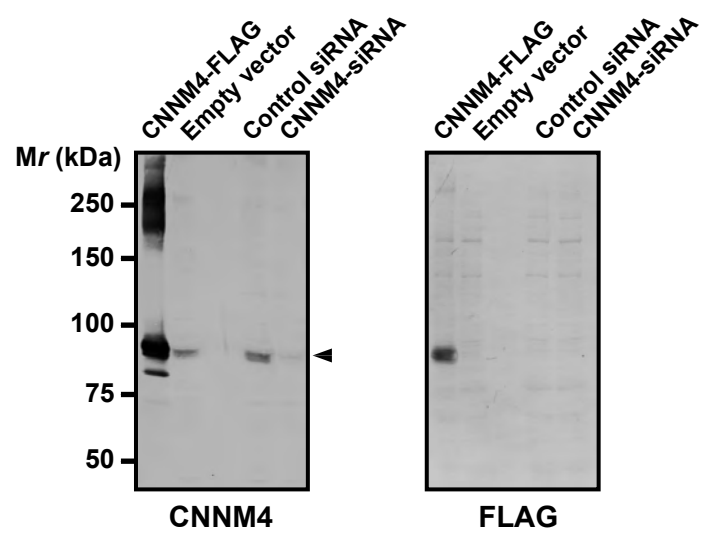

**Figure S1**

Supplement: Figure S1 — Characterization of the anti-CNNM4 antibody. Lysates of HEK293 cells transfected with CNNM4-FLAG or CNNM4-siRNA were subjected to immunoblotting analyses with the anti-CNNM4 antibody. The endogenous CNNM4 signal in HEK293 cells is indicated with an arrowhead. (PDF) [file pgen.1003983.s001.pdf]

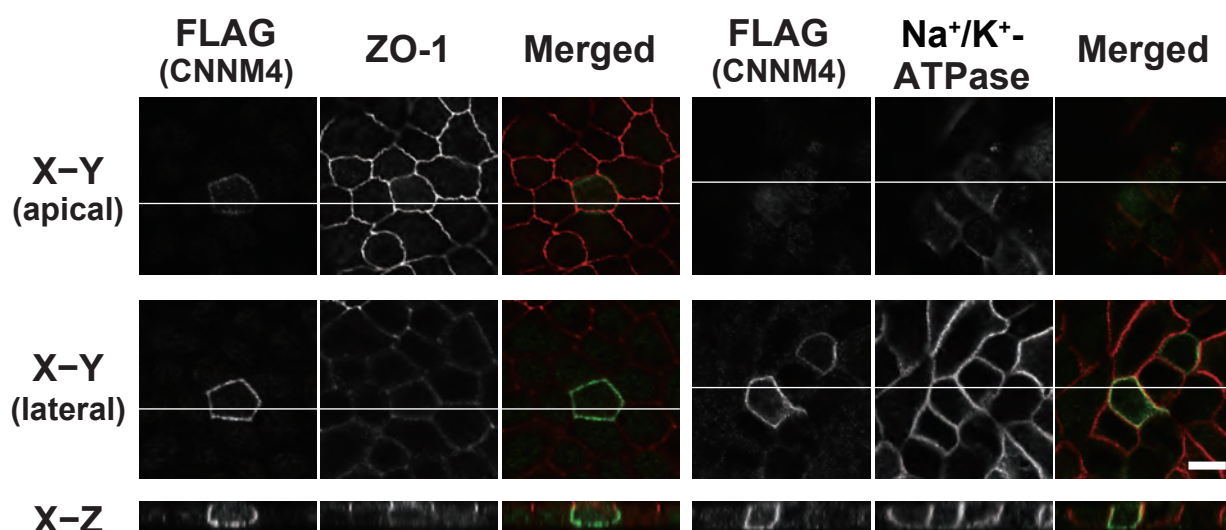

**Figure S2**

Supplement: Figure S2 — Basolateral localization of ectopically expressed CNNM4 in MDCK epithelial cells. MDCK cells transfected with CNNM4-FLAG were subjected to immunofluorescence staining with the antibodies for FLAG (green) and ZO-1 (red) or Na+/K+ ATPase (red). Horizontal section images (X–Y), at the level of the apical and lateral membranes, and vertically reconstituted images (X–Z) are shown. Vertical sections were taken at the white lines indicated in the X–Y images. Bar, 10 µm. (PDF) [file pgen.1003983.s002.pdf]

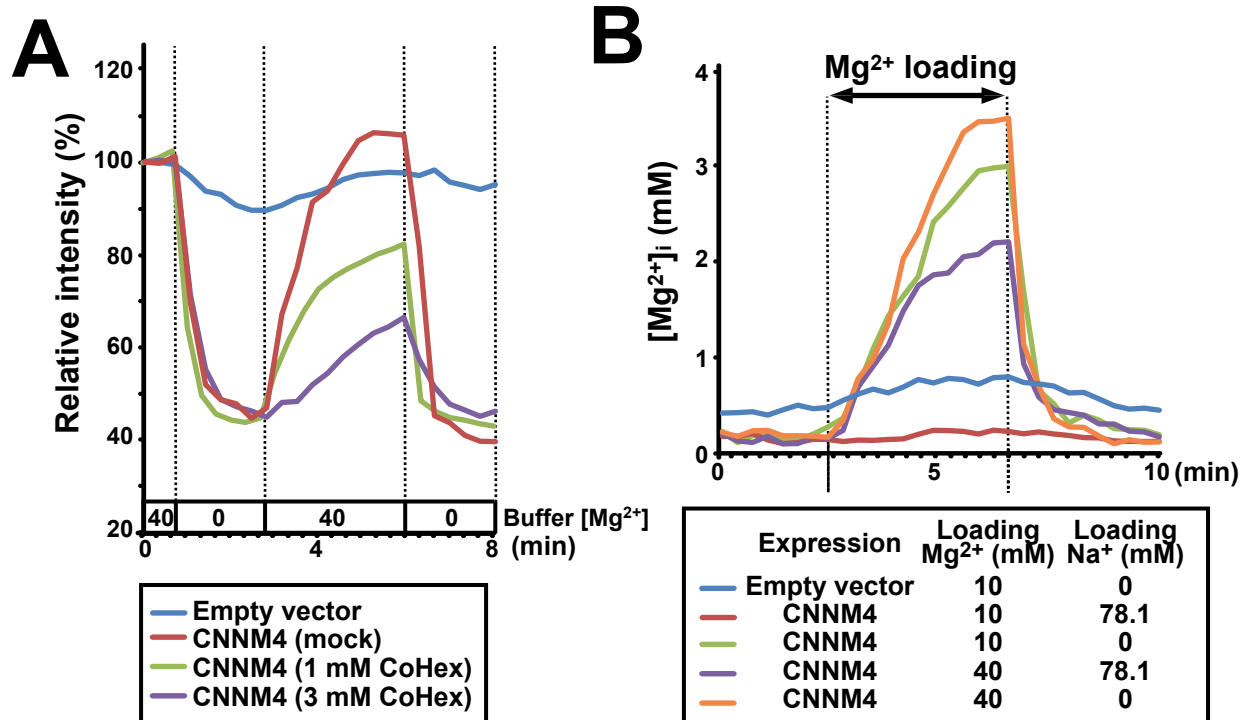

**Figure S3**

Supplement: Figure S3 — Characterization of Mg2+ influx in CNNM4-expressing cells. (A) HEK293 cells expressing CNNM4-FLAG were loaded with Magnesium Green and then subjected to time-lapse imaging analyses in the presence of 1 or 3 mM CoHex, under various extracellular solutions. The Mg2+ concentration in the extracellular solution is indicated. The means of relative fluorescence intensities of 10 cells are indicated. (B) HEK293 cells expressing CNNM4-FLAG were loaded with Mag-fura2, and then subjected to Mg2+ loading assays. The cells were incubated with extracellular solutions containing various concentrations of Mg2+ and Na+ during the indicated period (“Mg2+ loading”). The Mg2+ and Na+ concentrations in the extracellular solutions during the loading period, and the means of [Mg2+]i of 4 independent experiments (10 cells for each experiment) are indicated. See Materials and Methods for details. (PDF) [file pgen.1003983.s003.pdf]

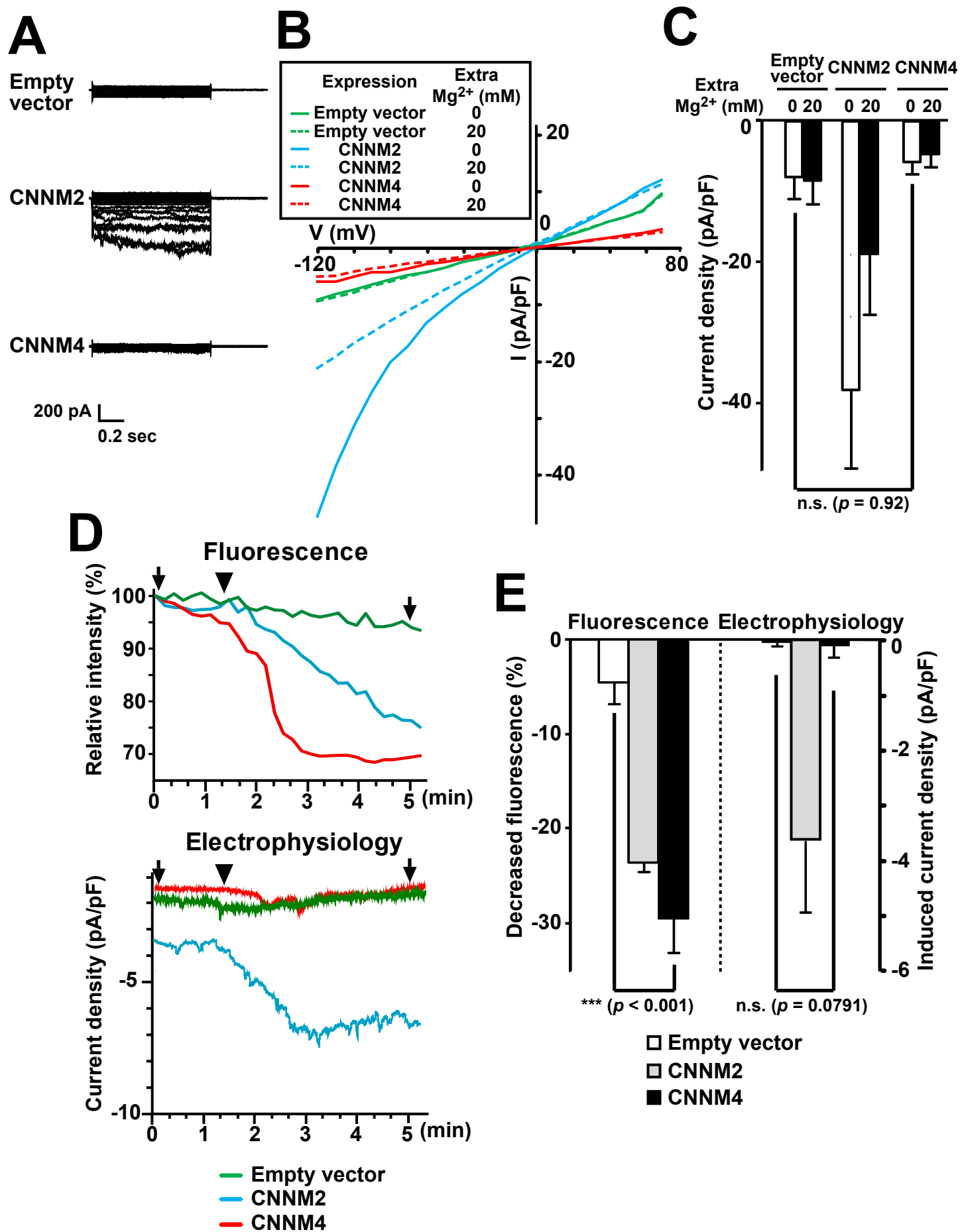

**Figure S4**

Supplement: Figure S4 — Electrophysiological recordings. (A) HEK293 cells transfected with the indicated expression constructs were subjected to electrophysiological recordings under whole-cell configuration. The cells were voltage-clamped between −120 mV and 70 mV in steps of 10 mV, and the representative traces of each cell are indicated. (B) Average I–V relationship of control (green), CNNM2- (blue), or CNNM4- (red) expressing cells recorded either in the presence (dotted lines) or absence (solid lines) of extracellular Mg2+ (n = 5–6). (C) Current densities at −110 mV recorded either in the presence or absence of extracellular Mg2+. Data are presented as mean ± s.e.m. of 5–6 cells. p value was determined by Student's two-tailed t-test. n.s.: not significant. (D) HEK293 cells transfected with the indicated constructs were subjected to simultaneous Mg2+ imaging and electrophysiological recording experiments. The extracellular solution was changed from Mg2+-containing to an Mg2+-free solution at the time point indicated by arrowheads. Means of relative fluorescence intensities and current densities (at −10 mV) of 4–6 cells are indicated. (E) Decreased fluorescence intensities and induced current densities were observed during the period between the arrows in (D). Data are presented as mean ± s.e.m. of 4–6 cells. p values were determined by Student's two-tailed t-test. ***p<0.001, n.s.: not significant. (PDF) [file pgen.1003983.s004.pdf]

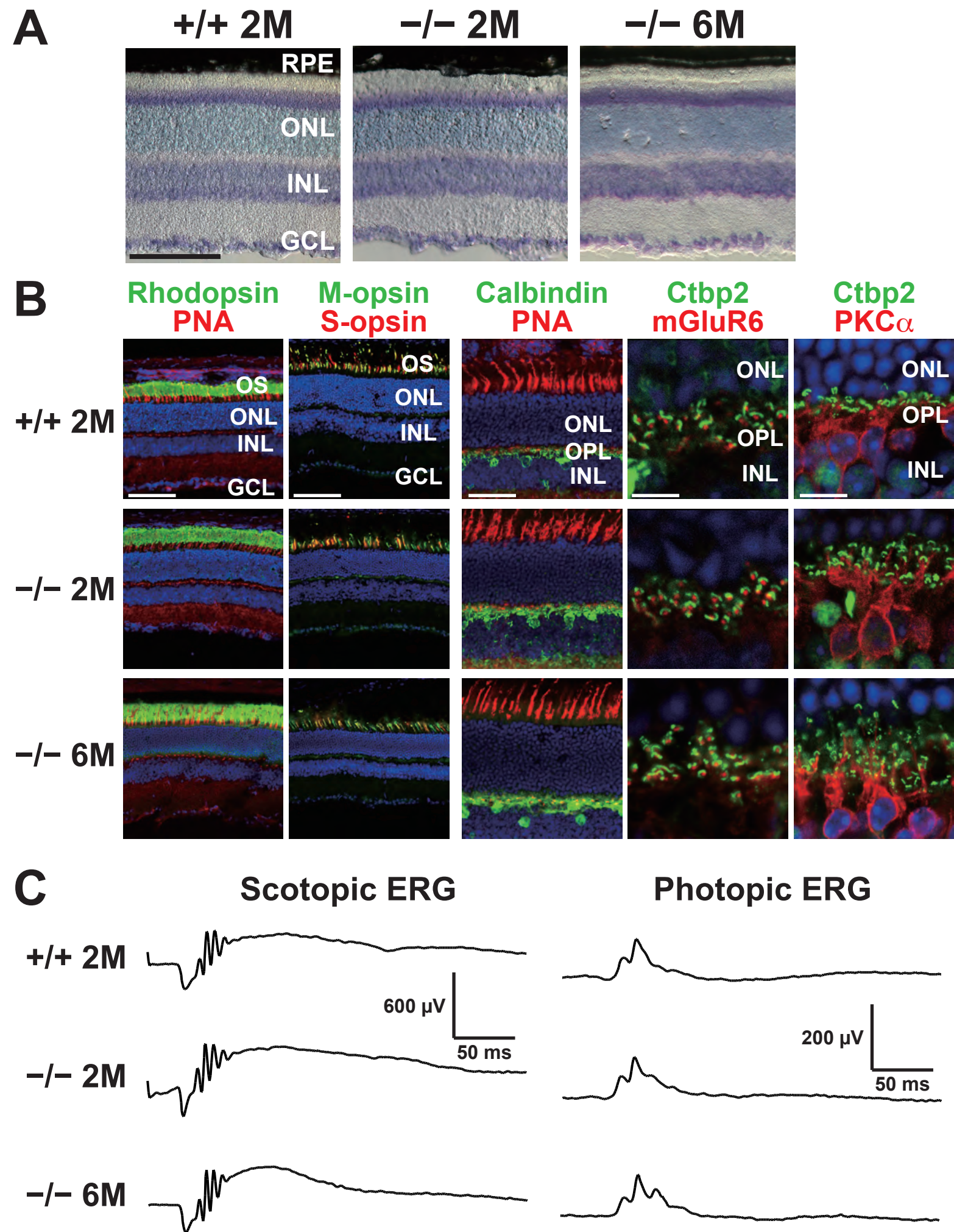

**Figure S5**

Supplement: Figure S5 — Histological and ERG analysis of the retina. (A) Retinal sections from 2- and 6-month old CNNM4+/+ and CNNM4−/− mice were stained with toluidine blue. Bar, 100 µm. (B) Retinal sections were stained with the indicated antibodies or lectins. Bars, 100 µm (Rhodopsin/PNA; M-opsin/S-opsin); 50 µm (Calbindin/PNA); 10 µm (Ctbp2/mGluR6; Ctbp2/PKCα). (C) Representative ERG waveforms recorded from 2- and 6-month old CNNM4+/+ and CNNM4−/− mice. Scotopic and photopic ERGs with stroboscopic stimuli of 1.0 log cd-s/m2 are shown. GCL, ganglion cell layer; INL, inner nuclear layer; ONL, outer nuclear layer; OPL, outer plexiform layer; OS, outer segment; RPE, retinal pigment epithelium. (PDF) [file pgen.1003983.s005.pdf]
